# Identification and characterization of *Colletotrichum* species causing apple bitter rot in New York and description of *C. noveboracense* sp. nov.

**DOI:** 10.1038/s41598-020-66761-9

**Published:** 2020-07-06

**Authors:** Fatemeh Khodadadi, Jonathan B. González, Phillip L. Martin, Emily Giroux, Guillaume J. Bilodeau, Kari A. Peter, Vinson P. Doyle, Srđan G. Aćimović

**Affiliations:** 1 Cornell University, Plant Pathology and Plant-Microbe Biology Section, School of Integrative Plant Science, Hudson Valley Research Laboratory, Highland, NY USA; 2 Cornell University, Plant Pathology and Plant-Microbe Biology Section, School of Integrative Plant Science, Ithaca, NY USA; 3 Pennsylvania State University, Department of Plant Pathology and Environmental Microbiology, Fruit Research and Extension Center, Biglerville, PA USA; 4 Pathogen Identification Research Laboratory, Canadian Food Inspection Agency, Ottawa, Ontario, Canada; 5 Louisiana State University AgCenter, Department of Plant Pathology and Crop Physiology, Baton Rouge, Louisiana USA

**Keywords:** Molecular biology, Plant sciences, Fungi, Pathogens, Fungal genes

## Abstract

Apple bitter rot caused by *Colletotrichum* species is a growing problem worldwide. *Colletotrichum* spp. are economically important but taxonomically un-resolved. Identification of *Colletotrichum* spp. is critical due to potential species-level differences in pathogenicity-related characteristics. A 400-isolate collection from New York apple orchards were morphologically assorted to two groups, *C. acutatum* species complex (*CASC*) and *C. gloeosporioides* species complex (*CGSC*). A sub-sample of 44 representative isolates, spanning the geographical distribution and apple varieties, were assigned to species based on multi-locus phylogenetic analyses of *nrITS, GAPDH* and *TUB2* for *CASC*, and *ITS, GAPDH, CAL, ACT, TUB2, APN2, ApMat* and *GS* genes for *CGSC*. The dominant species was *C. fioriniae*, followed by *C. chrysophilum* and a novel species, *C. noveboracense*, described in this study. This study represents the first report of *C. chrysophilum* and *C. noveboracense* as pathogens of apple. We assessed the enzyme activity and fungicide sensitivity for isolates identified in New York. All isolates showed amylolytic, cellulolytic and lipolytic, but not proteolytic activity. *C. chrysophilum* showed the highest cellulase and the lowest lipase activity, while *C. noveboracense* had the highest amylase activity. Fungicide assays showed that *C. fioriniae* was sensitive to benzovindiflupyr and thiabendazole, while *C. chrysophilum* and *C. noveboracense* were sensitive to fludioxonil, pyraclostrobin and difenoconazole. All species were pathogenic on apple fruit with varying lesion sizes. Our findings of differing pathogenicity-related characteristics among the three species demonstrate the importance of accurate species identification for any downstream investigations of *Colletotrichum* spp. in major apple growing regions.

## Introduction

*Colletotrichum* is a cosmopolitan fungal genus comprised of more than 189 species distributed throughout tropical and temperate regions worldwide^1–3^*. Colletotrichum* species cause devastating diseases such as anthracnose and fruit rots on a broad range of plant hosts^4,5^ and affect valuable fruit crops such as banana, strawberry, citrus, avocado, papaya, mango and apple^6–10^.

Apple (*Malus domestica* Borkh.), native to central Asia and then introduced to the west and other parts of the world^11,12^, is a major fruit crop cultivated in temperate regions today. The United States produces about five million metric tons on almost 130,309 hectares, making it the second largest apple producer after China^13^. In the U.S. in 2018, apples were grown commercially in 20 states with New York ranking as the second largest producer^14^, with 17 thousand hectares of apples cultivated for over 260 million dollars in value^15^.

Apple is vulnerable to a wide range of diseases affecting yield and fruit quality. Bitter rot, caused by *Colletotrichum* spp., is one of the most important fungal diseases of apple causing remarkable economic losses under wet and warm weather conditions in the US and globally^6,16,17^. Reports of apple fruit losses to bitter rot in New York range from 14–25%^18,19^ and up to 100% in organic orchards, reach up to 100% in North Carolina^20^ and are 30% on average in Kentucky, where some orchards were a complete loss^21,22^. Among the nine major clades of *Colletotrichum*, the *C. gloeosporioides* species complex (*CGSC*) and the *C. acutatum* species complex (*CASC*) are the two most common clades that cause bitter rot of apple^2,16,17^. *C. fioriniae*, *C. nymphaea* and *C. godetiae*^23^ from *CASC* and *C. fructicola, C. aenigma, C. siamense* and *C. theobromicola* from *CGSC*^3,17,24–26^, are known so far to cause bitter rot on apple worldwide. Besides causing bitter rot, species such as *C. limetticola, C. paranaense, C. melonis* in *CASC* and *C. fructicola* in *CGSC* cause Glomerella leaf spot (GLS) of apple^27^. Although these two diseases are associated with the same fungal genus, differences in pathogenicity, morphology and cultural characteristics of species have been reported^28^.

Accurate identification of *Colletotrichum* species causing bitter rot is crucial due to potential species-level variation in pathogenicity-related characteristics. Identifying the causal agent(s) facilitates resistance breeding programs and determines the best control strategies for apple diseases^29,30^. Identification of *Colletotrichum* to the species level was traditionally reliant on host, cultural and morphological descriptions, as well as comparison of nuclear rDNA internal transcribed spacer (*ITS1-5.8S-ITS2* = *ITS*) sequences^31–35^. However, these identification techniques are limited in their effectiveness as growth medium and temperature are known to cause variation in cultural and morphological characteristics, such as size and shape of conidia, colony growth rate and pigmentation of *Colletotrichum* isolates^3,29,34^. The ITS regions known as the barcode locus for fungi^36,37^, is considered insufficient to delimit species in the *CGSC*^37–39^.

While species delimitation using morphology and *ITS*-based phylogeny remains insufficient for resolution of *Colletotrichum* at the species level, multi-locus phylogenetic analyses have proven reliable in addressing challenges in the identification of *Colletotrichum* species^3,10,40–43^. In addition to *ITS*, loci such as glutamine synthetase (*GS*), glyceraldehyde-3-phosphate dehydrogenase (*GAPDH*), calmodulin (*CAL*), actin (*ACT*), chitin synthase (*CHS–1*), β-tubulin (*TUB2*), DNA lyase (*APN2*), and the intergenic region between DNA lyase and the mating type (Mat1-2) gene (*ApMat*) have been used to resolve various species in the *CGSC*^3,26,41,44–53^.

Accurate identification of *Colletotrichum* species causing bitter rot is a prerequisite to successfully manage this disease in apple production regions because different species of *Colletotrichum* respond differently to fungicides and vary in traits such as enzyme activity and pathogenicity^17,54,55^. Accordingly, characterizing fungicide sensitivity, enzyme activity and pathogenicity of species is of extreme importance for future research on control of bitter rot. The ability of *Colletotrichum* species to produce extracellular enzymes determines their pathogenicity and virulence capacity^34^. The variable levels of amylolytic, pectolytic, polymethylgalacturonase (PMG) and polygalacturonase (PG) activity was detected in *Colletotrichum* species associated with different plant diseases^55–59^.

Several studies showed variable fungicide sensitivity within and between the two main complexes causing bitter rot, *CASC* and *CGSC*^25,60–62^, which gives rise to challenges in bitter rot management^24,34,63,64^. For instance, a response variation among *C. acutatum* and *C. gloeosporioides* isolates collected in Kentucky was observed following the *in vitro* evaluation of fungicides^17^. Within each complex, The *in vitro* screening to determine the half maximal effective concentration (EC_50_) allow tracking the sensitivity of *Colletotrichum* species to fungicides and manage the risk of fungicide resistance as a rising problem^65–67^, which to some extent stems from applying ineffective concentrations of fungicides^68^.

In this study we aimed to (1) Identify the *Colletotrichum* species causing bitter rot of apple in New York; (2) Determine *in vitro* enzyme activity of *Colletotrichum* species; (3) Evaluate sensitivity of collected *Colletotrichum* species to several key fungicides; and (4) Compare the pathogenicity of these species on apple fruit.

## Results

### Screening of isolates

In 2017 and 2018, we collected a total of 400 *Colletotrichum* isolates from apple fruit in New York and other states. Isolates were morphologically screened for colony color, growth rate, sporulation capacity and conidial shape on PDA, and organized into two general morphological types. The first morphotype, comprising more than 60% of isolates, included isolates producing a distinct salmon to red colony color, fusiform spores, and slower growth rate on PDA. The second morphotype included two distinct groups: 12.5% isolates producing colonies with mycelia ranging from dominantly white to off-white with slightly light grey centers and rare sporulation; and 25% isolates with light to dark gray mycelium, cylindrical rounded spores and faster growth rate on PDA. The characteristics of isolates in the morphotype 1 and 2 were consistent with the descriptions of *CASC* and *CGSC*, respectively. Initially, 100 isolates from morphotypes 1 and 2, representing the morphological variation, geographical and apple cultivar range, were selected for species identification using *ITS*, *TUB2* and *GAPDH* sequences. Facing challenges in species delimitation within *CGSC*, we increased the number of partially sequenced genes to eight for isolates belonging to *CGSC*, adding *ACT*, *ApMat*, *CAL*, *GS* and *APN2*, and reduced the number of isolates for identification and downstream analyses to 44 (19 from morphotype 1 and 25 from morphotype 2).

### Multiplex PCR assay

An amplicon of approximately 349 bp was recovered using *Colletotrichum*-specific *GAPDH* primers GDF1/C-GAPDH-R, confirming that all isolates were *Colletotrichum* spp. The *CAL* gene primers specific to species complex confirmed that of the 44 isolates, 19 from morphotype 1 were members of the *CASC* and 25 from morphotype 2 were members of the *CGSC* (491 and 649 bp amplicons respectively; Supplementary Fig. S1). Amplified fragments of expected lengths representing the *CASC* and *CGSC* support^49^ that the *GAPDH*/*CAL* multiplex PCR approach is satisfactory at differentiating these two species complexes.

### Phylogenetic analyses

**Figure 1:**
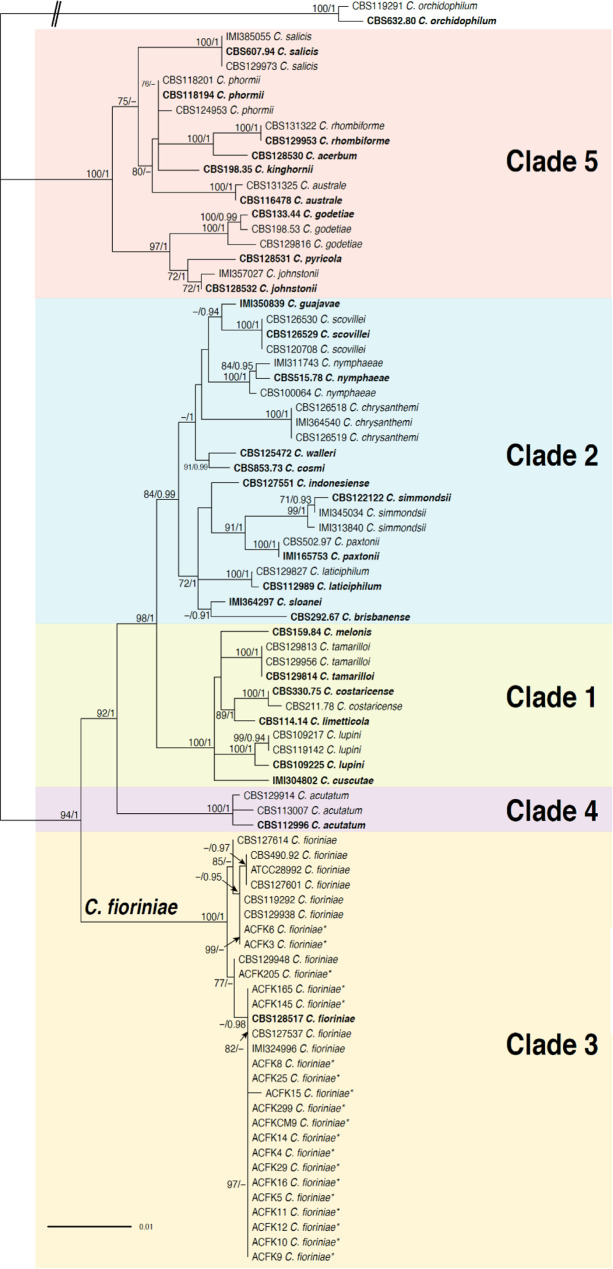
Maximum-likelihood phylogeny inferred from *ITS*, *TUB2* and *GAPDH* sequences from the *Colletotrichum acutatum* species complex. *Colletotrichum* isolates from this study are denoted with asterisks. The remaining taxa are reference isolates retrieved from NCBI. The phylogeny is rooted with *C*. *orchidophilum* (CBS 119291) and *C*. *orchidophilum* (CBS 632.80) as the outgroup. Bootstrap support values greater than 70 and posterior probabilities greater than 0.90 are shown on the branches (BS/PP). Type isolates are in bold font. Double hash marks indicate branch lengths shortened at least 2-fold to facilitate visualization. Scale bar represents the estimated number of substitutions per site.

The *ITS* phylogeny concurred with the multiplex PCR assay in that *Colletotrichum* isolates collected in this study fell into two *Colletotrichum* species complexes: *C*. *acutatum* (19 isolates) and *C. gloeosporioides* (25 isolates) with high support (Supplementary Fig. S2). The *C. acutatum* phylogeny dataset included 85 taxa (including 19 isolates from this study) and 1278 characters consisting of three loci (*ITS*, *TUB2* and *GAPDH*). Two *C*. *orchidophilum* isolates, CBS 119291, and CBS 632.80, were used as an outgroup. All five major *C. acutatum* clades^42^ were resolved with high support (BS ≥ 84, PP = 0.99; Fig. 1). Both Bayesian Inference (BI) and Maximum Likelihood (ML) analyses revealed that the 19 isolates collected in this study clustered with *C. fioriniae* as part of clade 3 of the *CASC* with full support (BS/PP: 100/1; Fig. 1) and are hereafter designated as *C. fioriniae*. We found that the majority of isolates in this study included in the *CASC* phylogentic analysis clustered with *C. fioriniae* type isolate CBS 128517, with high PP support (0.98), but lacking BS support ≥ 70. The remaining three isolates (ACFK3, ACFK6, ACFK205) fell outside of this group (Fig. 1), though remaining within the highly supported Clade 3 (*C. fioriniae*). ACFK3 and ACFK6 were placed well-within a different subclade with high BS support (85) but lacking PP support ≥ 0.90.While further analysis is required, we believe this separation of the isolates in this study may be similar to the previous fnding that the *C. fioriniae* clade is partitioned into two major subclades^42^.

The *C. gloeosporioides* phylogeny dataset included 201 taxa (including 25 isolates collected in this study and Coll940) and 4890 characters consisting of eight loci (*ACT, ApMat, CAL, GAPDH, GS, APN2, ITS* and *TUB2*). The outgroup included one member of the *CASC*, *C. javanense* CBS 144963, and two members of the *C. boninense* complex, *C. boninense* CBS 123755 and *C. hippeastri* ICMP17920.

**Figure 2:**
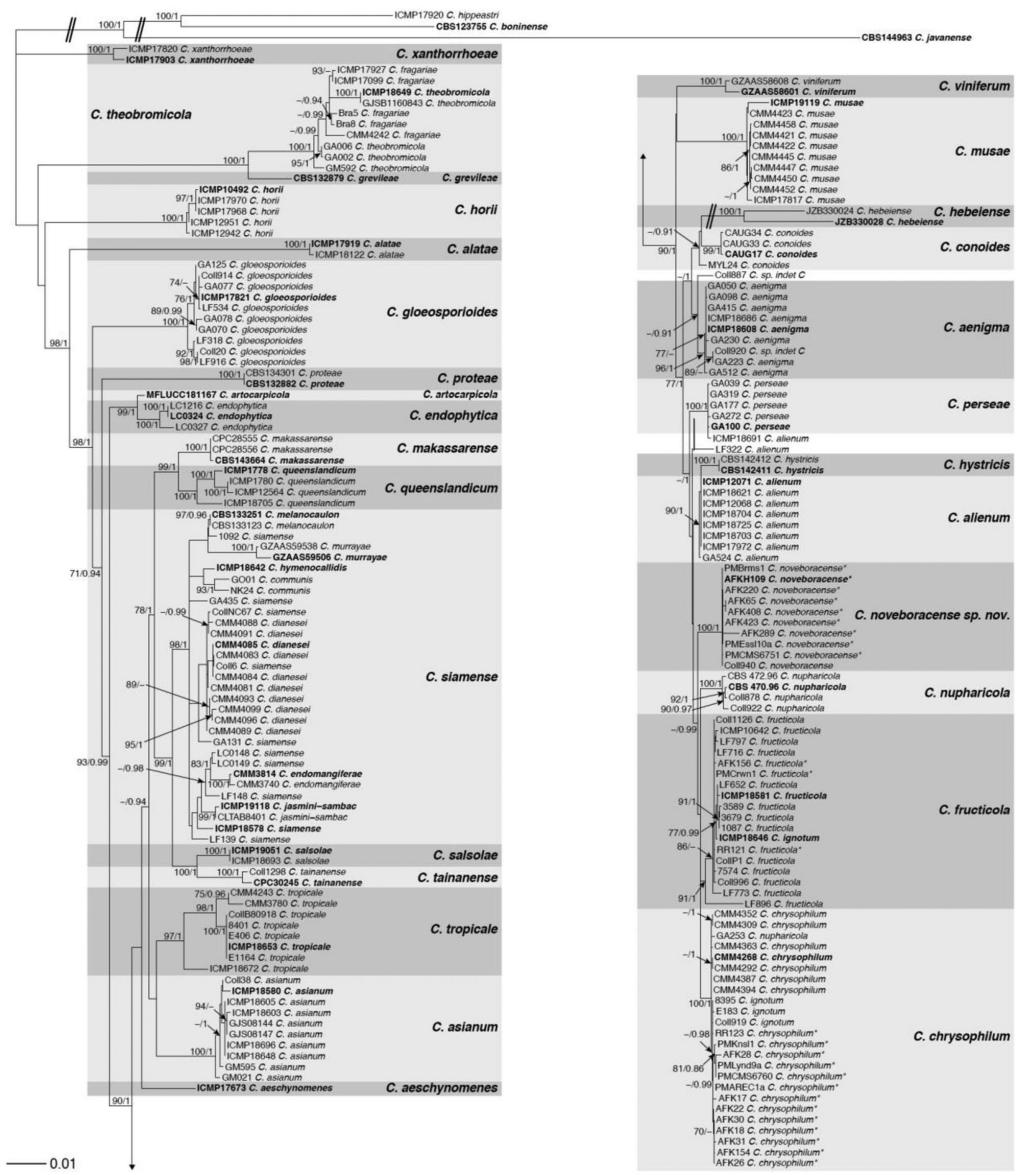
Maximum-likelihood phylogeny inferred from eight loci (*ACT, CAL, GAPDH, GS, ITS, ApMat, APN2* and *TUB2*) from the *Colletotrichum gloeosporioides* species complex. *Colletotrichum* isolates from this study are denoted with asterisks. The remaining taxa are reference isolates retrieved from NCBI. The phylogeny is rooted with *C. javanense* CBS 144963, *C. boninense* CBS 123755 and *C. hippeastri* ICMP17920 as the outgroup. Bootstrap support values greater than 70 and posterior probabilities greater than 0.90 are shown on the branches (BS/PP). Type isolates are in bold font. Double hash marks indicate branch lengths shortened at least 2-fold to facilitate visualization. Scale bar represents the estimated number of substitutions per site.

The 26 isolates belonging to *CGSC* collected in this study from apple in New York, Virginia, and Pennsylvania, were found to group into three distinct clades, two of which represent previously described species within the *CGSC*. Twelve isolates, AFK17, AFK18, AFK22, AFK26, AFK28, AFK30 and AFK31 from New York, isolates AFK154 and PMAREC-1a from Virginia, and isolates PMKnsl-1, PMCMS-6760 and PMLynd-9a from Pennsylvania, grouped with the ex-type strain of *C. chrysophilum* with maximum support (BS/PP: 100/1; Fig. 2) and are hereafter designated as *C. chrysophilum*. To our knowledge, this is the first time that the *C. chrysophilum* species has been reported to cause bitter rot disease on apple. After *C. fioriniae*, *C. chrysophilum* was the second most abundant species causing bitter rot disease in New York.

Two isolates, AFK156 and PMCrwn1 from Virginia, grouped with the ex-type strain of *C. fructicola* (BS/PP: 91/1; Fig. 2) and are hereafter designated as *C. fructicola*. Of the two *CGSC* isolates originally collected from peach in South Carolina^49^, RR12-1 was found to group with *C. fructicola*, as previously reported^47^. The second isolate, RR12-3, previously recognized as *C. fructicola* using a multi-locus analysis (*CAL, GAPDH* and *TUB2*^49^), was found clustered within the fully supported *C. chrysophilum* clade in our eight-gene multi-locus analysis (Fig. 2). Further, *CGSC* member GA253, isolated from avocado in Israel^69^, which was previously identified as *C. nupharicola* using an *ApMat* phylogeny as well as a six-gene multi-locus analysis^69^, was found to cluster within the *C. chrysophilum* clade (Fig. 2). No isolates belonging to *C. fructicola* were identified from apple fruit in New York and Pennsylvania.

The remaining 9 isolates, AFKH109, AFK65, AFK220, AFK289, AFK408 and AFK423 from New York and PMEssl-10a, PMCMS-6751 and PMBrms-1 from Pennsylvania, formed a separate, distinct clade with maximum support and independent from any recognized species in the *CGSC* (BS/PP: 100/1; Fig. 2). This distinct clade included isolate Coll940, which was originally isolated from leaves of black walnut (*Juglans nigra*) in Oklahoma and had an uncertain placement based on *nrITS*, *TUB2*, *APN2* and *ApMat* analyses^53^. We pursued further analyses to determine if this unique cluster represented a new, undescribed lineage in the *CGSC*. For phylogenetic models and partitioning schemes see Supplementary Table S1.

### Species delimitation

All *Colletotrichum* isolates from apple were assigned to a lineage containing the ex-type of a previously described species using genealogical concordance phylogenetic species recognition approach (GCPSR) except for AFKH109, AFK65, AFK220, AFK289, AFK408, AFK423, PMEssl-10a, PMCMS-6751, and PMBrms-1. These isolates were strongly supported in the 8-locus concatenated analyses as monophyletic (BS = 100; PP = 1) and sister to *C. fructicola, C. nupharicola* and *C. chrysophilum*. Among the independent gene trees, these isolates were strongly supported as monophyletic in the *ApMat* (BS = 99; PP = 1), *APN2* (BS = 100; PP = 1), and *GS* (BS = 92; PP = 1) phylogenies. These isolates were also inferred to be monophyletic in the *ACT* phylogeny, although with weak support in both the ML analysis (BS = 54) and BI analysis (PP = 0.85). While they were not monophyletic in the phylogenies inferred from *TUB2*, *ITS*, *GAPDH* and *CAL*, there was no strongly supported conflict in those trees. Our results are consistent with the criteria of GCPSR for recognizing these isolates as an independent lineage representing a novel species of *Colletotrichum*, named as *C. noveboracense*. Phylogenetic models and partitioning schemes used can be found in Supplementary Table S1.

### Morphology characterization

**Figure 3:**
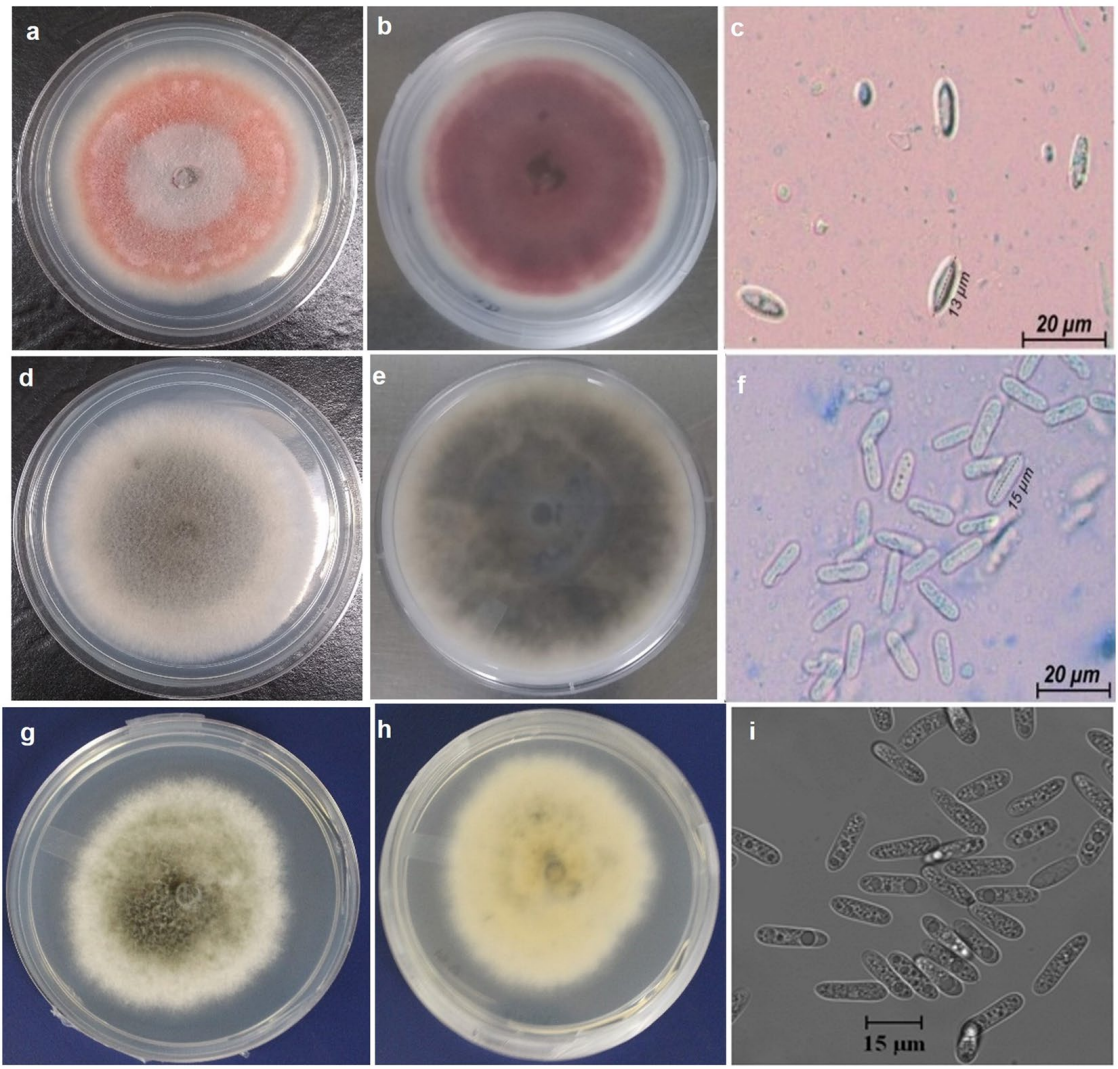
Morphological characteristics (colony color and conidial shape) of *Colletotrichum* spp. isolated from bitter rot-infected apple fruit. Colony color of *Colletotrichum fioriniae:* (**a**) Front, (**b**) Reverse, (**c**) Conidial shape; colony color of *Colletotrichum chrysophilum:* (**d**) Front, (**e**) Reverse, (**f**) Conidial shape; Colony color of *Colletotrichum fructicola* (isolate AFK156 from Virginia): (**g**) Front, (**h**) Reverse, (**i**) Conidial shape.

We described morphological characteristics including colony color, conidial shape, measurements of colony growth rate and conidial length and width for several isolates of each *Colletotrichum* species causing apple bitter rot in this study (*C. fioriniae, C. chrysophilum, C. fructicola* and *C. noveboracense*). The isolates of *C. fioriniae* produced salmon to red conidial masses on 7-day-old cultures on PDA in both front and reverse sides and produced fusiform conidia after 10 days on PDA (Fig. 3a–c). Isolates belonging to *C. chrysophilum* initially presented colonies in white to light gray and progressively turned to dark grey in the center covered with predominantly black acervuli, producing orange conidial masses with longer incubation time. Cylindrical conidia with rounded ends developed after 10 days of incubation on PDA for this species (Fig. 3d–f). *C. fructicola* formed off-white to slightly gray aerial mycelium and yellowish to grey in reverse, developing cylindrical conidia with rounded ends after 10 days of incubation on PDA (Fig. 3g–i). Comparisons of conidial dimensions and shape, colony growth rates, as well as the description of colony color are presented in Table 1 Detailed morphological description for *C. noveboracense* is provided in the Taxonomy section.

**Table 1:** Morphological characteristics of *Colletotrichum* species in this study.

Characteristics	Species			
	*C. fioriniae*	*C. noveboracense*	*C. fructicola*	*C. chrysophilum*
Colony color	Initially white then covered with pink to salmon conidial masses. reverse pink to red	Predominantly white mycelial masses; reverse off-white to white	white mycelia with grey to dark grey at the center; reverse yellowish yellow	Light to dark gray mycelium; reverse: dark grey to white in the margins
Conidium length (μm) $\bar{{\rm{x}}}$ ± SD**	11.43 ± 1.41a (11–16.1) n = 25	13.6 ± 0.86b* (12.1–15.6) n = 25	16.6 ± 1.83c (13.2–20.3) n = 25	14 ± 1.46b (11.7–17.6) n = 25
Conidium width (μm) $\bar{{\rm{x}}}$ ± SD	4.38 ± 0.7a (3.2–5.6) n = 25	5.7 ± 0.48b (4.6–6.4) n = 25	5.10 ± 0.44c (4.4–5.9) n = 25	4.9 ± 0.64c (4–6.3) n = 25
Conidium shape	Fusiform with pointed ends	Cylindrical	Cylindrical with both ends rounded	Cylindrical with rounded ends
Growth rate (mm/day) $\bar{{\rm{x}}}$ ± SD	7.89 ± 0.75a (4–11.1)	13.10 ± 1.30b (7–17.5)	13.1 ± 0.16b (7–17)	14.95 ± 0.79c (12–20)

*Values followed by different letter were significantly different (*P* < 0.05).

**SD = Standard deviation.

### TAXONOMY

***Colletotrichum noveboracense*** F. Khodadadi, P. L. Martin, V. P. Doyle, J. B. González and S. G. Aćimović, sp. nov.

**Figure 4:**
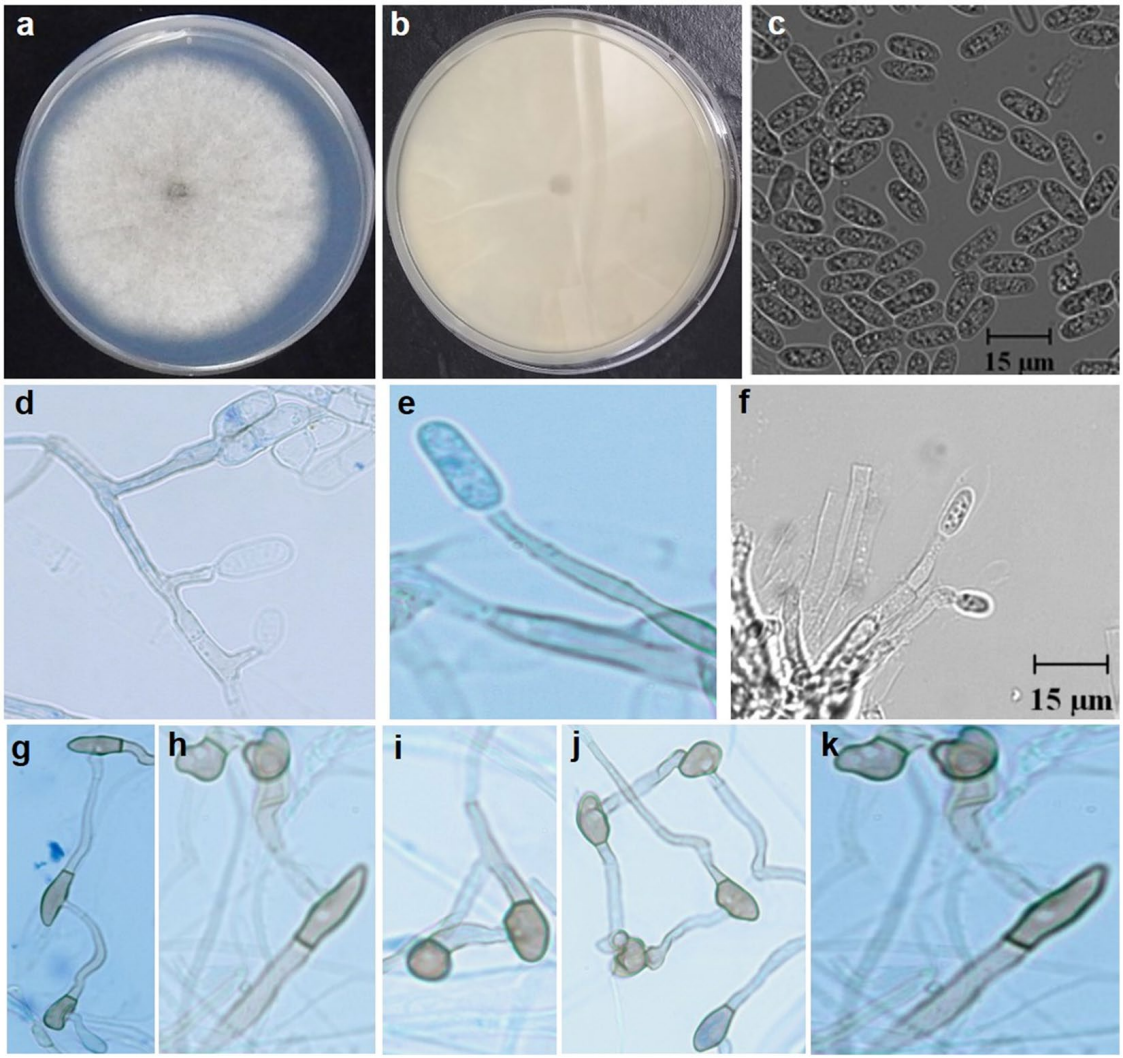
*Colletotrichum noveboracense* (CBS 146410, ex-holotype culture). (**a**) Colony on PDA, (**b**) Reverse side of the colony on PDA, (**c**) Conidia on ½ strength PDA, (**d–f**) Conidiophores and conidia, (**g–k**) Appressoria. All scale bars = 15 µm.

**Figure 5:**
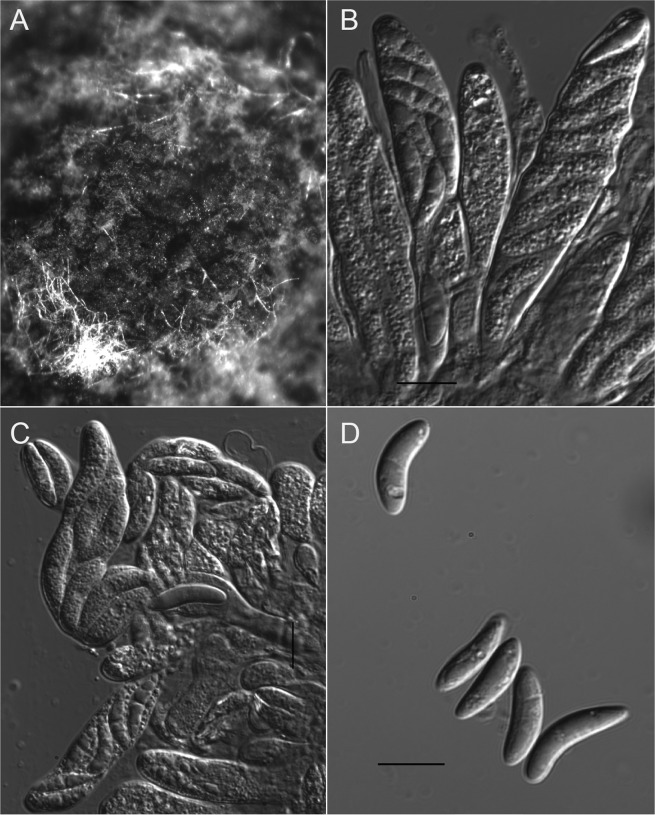
Teleomorph morphology of *Colletotrichum noveboracense* (CBS 146410, Ex-holotype culture) on OMA overlaid with filter paper. (**A**) Clustered perithecia, (**B, C**) Asci, (**D**) Ascospores, All scale bars = 10 µm.

MycoBank MB 833232 (Figs. 4 and 5)

### Etymology

The specific epithet is a combination of the long-established Latin name for New York (Noveboracum) state in the United States of America and the Latin -ensis, denoting the origin of the holotype.

### Holotype

The United States of America: New York State, Hudson, from fruit lesion of *Malus domestica* cultivar Idared. July. 2017, F. Khodadadi and S. G. Aćimović (CBS 146410; AFKH109 – holotype).

### Description

Growth rate on full strength PDA at 25 °C, 13.1 ± 1.3 mm/d (avg. ± std. dev.) and 11.9 ± 0.9 mm/d on ½ strength PDA. Colonies on PDA white with light gray toward the center, reverse white to pale off-white and slightly grey at the center. Aerial mycelium on PDA white to off-white and cottony. Colony on CMA nearly invisible. Acervuli not observed. Perithecia solitary to clustered on SNA and OMA, dark brown to black, globose to obpyriform; ascospores allantoid, light olive (13.1–)15.9–17.6–19.8(–22.4) × (3.8–)4.4–4.8–5.1(–5.5) μm, length-width ratio (2.7–)3.4–3.7–4.0(–4.6). Conidiophores hyaline, smooth-walled, aseptate, unbranched. Conidiogenous cells hyaline, smooth-walled, cylindrical to ampulliform, monophialidic, often extending percurrently to form new monophialides and conidiogenous loci. Conidia formed from conidiogenous cells, one-celled, smooth-walled, hyaline, and cylindrical, sometimes oblong, contents appearing granular with occasional oil droplets. Conidia on ½ strength PDA (12.19–)12.5–13.03–14.9(–15.6) × (4.6–)4.8-5.3–5.9(–6.4) μm (avg. 13.6 × 5.7 μm, n = 25), length/width ratio (1.9–)2–2.3–2.7(–2.8) (avg. 2.41, n = 25). Appressoria (hyphopodia) in slide cultures, single, or in groups, light to medium brown, smooth-walled, oval, often with undulate margin (4.47–) 5.5–8.6–11.7(–13.5) × (3.7–)4.9–5.5–6.1(–6.2) μm (avg. 8.8 ± 2 × 4.9 ± 0.6 μm, n = 25). Hyphal diameter 1.8–4.5 μm.

### Habitat/host

Known from the states of New York and Pennsylvania, causing bitter rot on *Malus domestica* fruit and a single isolate from Oklahoma as a leaf endophyte on *Juglans nigra*^53^.

### Diagnosis

Isolates of *Colletotrichum noveboracense* are strongly supported as monophyletic by the combined analysis of *ACT*, *APN2*, *GS*, *CAL*, *ApMat*, *GAPDH*, *ITS* and *TUB2* and sister to *C. nupharicola*, *C. chrysophilum* and *C. fructicola*. *C. noveboracense* differs from *C. nupharicola* by having a faster growth rate on PDA as well as shorter and narrower conidia. *C. nupharicola* also differs in having an orange colony that turns black with age on PDA versus white to grey colony color for *C. noveboracense*. *C. noveboracense* differs from *C. fructicola* by having shorter conidia and lighter colonies on PDA and differs from *C. chrysophilum* by having a slower growth rate on PDA. Sequence data from *ApMat*, *APN2*, *GS* and *ACT* delimit* C. noveboracense*, but *C. noveboracense* could not be distinguished by sequences of *GAPDH*, *CAL*, *TUB2* and *ITS*.

### Additional specimens examined

USA. New York: Ulster County: on fruit of *Malus domestica*, Jul 2017, F. Khodadadi (AFK220, AFK408, AFK423, and AFK289); USA. New York: Colombia County: on fruit of *Malus domestica*, Jul 2017, F. Khodadadi (AFK65); USA. Pennsylvania: Adams County: on fruit of *Malus domestica*, late summer and fall of 2018, P. L. Martin (PMBrms-1); USA. Pennsylvania: Lehigh County: on fruit of *Malus domestica*, late summer and fall of 2018, P. L. Martin (PMCMS-6751); USA. Pennsylvania: Northumberland County: on fruit of *Malus domestica*, late summer and fall of 2018, P. L. Martin (PMEssl-10a); USA. Oklahoma: leaf endophyte of *Juglans nigra*, Cherokee County, 36.028725N, 95.185787W, June 10, 2010, V. Doyle Coll940.

### Notes

A very low sporulation rate was observed among the isolates collected from New York. No sporulation was seen on PDA and ½ strength PDA except for few isolates including AFKH109 which sparsely produced conidia on OMA. However, isolates collected from Pennsylvania sporulated on ½ strength PDA and OMA.

### Agar plate enzyme activity

**Figure 6:**
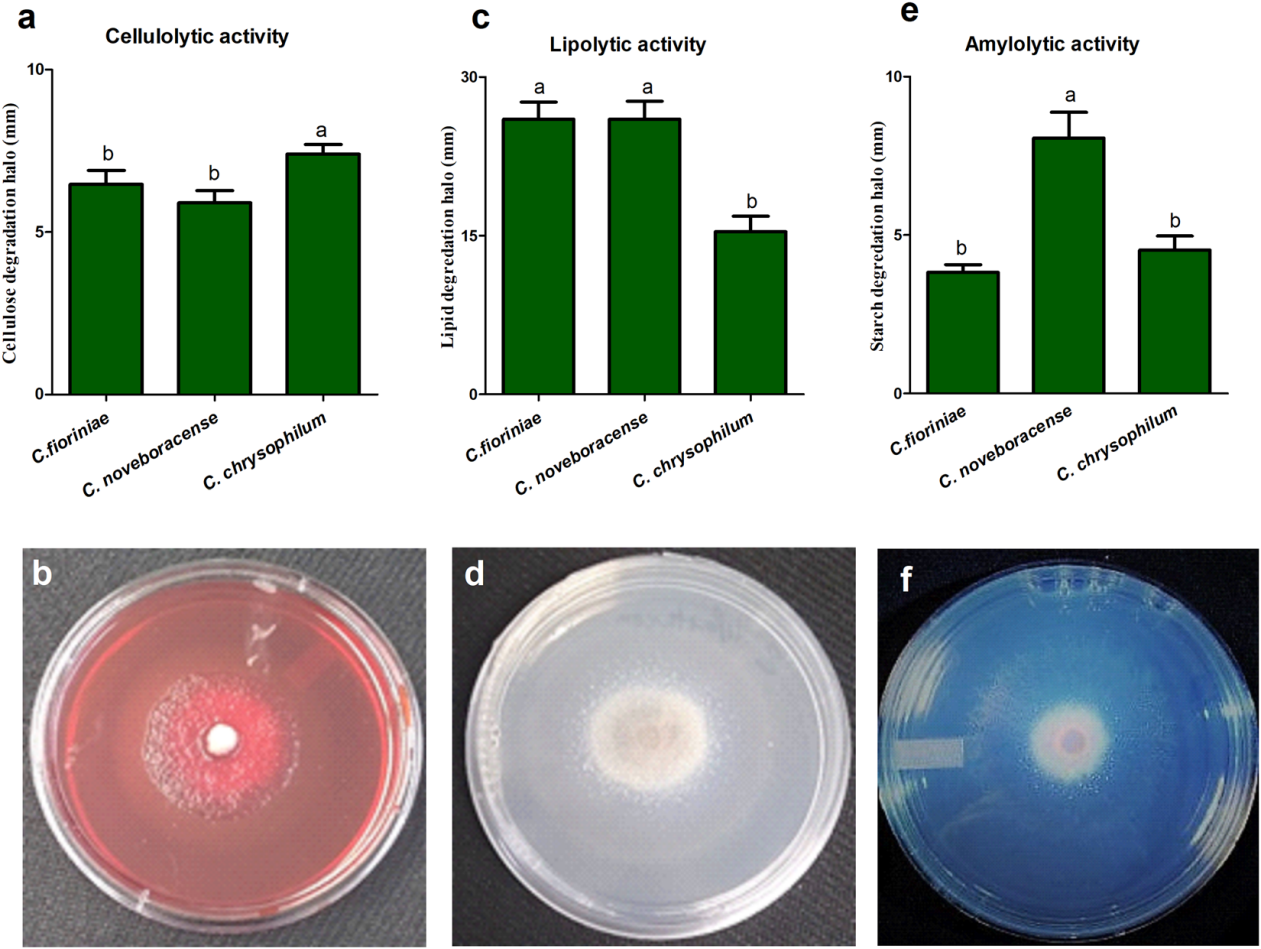
Qualitative enzyme activity of *Colletotrichum fioriniae*, *C. noveboracense* and *C. chrysophilum* isolates using the agar plate method at 5 days after incubation. (**a**) Mean cellulase degradation halo of *Colletotrichum* isolates; (**b**) Yellow halo formed after exposure to Congo red and fixation with NaCl; (**c**) Mean lipid degradation halo of *Colletotrichum* isolates; (**d**) White and clear halo representing lipolytic activity; (**e**) Mean starch degradation halo of *Colletotrichum* isolates; (**f**) Clear zone around the colony after exposure to iodine indicating amylolytic activity. *C. fioriniae* represented with isolates ACFK165, ACFK10, ACFK11, ACFK12, ACFK14, ACFK145, ACFK15, ACFK16, ACFK25, ACFK29, ACFK299, ACFK4, ACFK5, ACFK8, ACFK9, ACFKCM-9, ACFK205, ACFK3 and ACFK6; *C. noveboracense* represented with isolates AFK220, AFK289, AFK408, AFK423, AFKH109 and AFK65; and *C. chrysophilum* represented with isolates AFK17, AFK28, AFK154, AFK18, AFK22, AFK26, AFK30 and AFK31. Different letters indicate significant differences between species based on Bonferroni Comparison Posttest (*p* ≤ 0.05). Error bars represent standard deviation.

All isolates belonging to *C. noveboracense*, *C. fioriniae* and *C. chrysophilum* showed lipolytic, amylolytic and cellulolytic activity after five days of incubation on PDA (Fig. 6). However, none of the isolates showed halos of degradation for the proteolytic activity on skimmed milk. All isolates evaluated in this study showed cellulolytic activity as a yellow halo around the colony in plates including CMC stained with Congo red and secured with NaCl. *C. chrysophilum* showed a significantly larger mean degradation halo of 8 mm in cellulolytic activity assay when compared to *C. noveboracense* and *C. fioriniae* (mean halo zone 6 and 6.5 mm, respectively) (Fig. 6a,b). *Colletotrichum* isolates of all three species produced hallo around their colonies indicating their ability to produce lipase. *C. chrysophilum* isolates exhibited significantly the lowest lipid degradation with the mean halo diameter of 17 mm compared to isolates of *C. fioriniae* and *C. noveboracense*, with the mean halo diameter of 23 and 27 mm, respectively (Fig. 6c,d). The screening of *Colletotrichum* isolates on starch agar plates showed that all species produced halo zones reflecting amylase activity after exposure to iodine. The smallest and largest halo sizes for amylase belonged to *C. fioriniae* and *C. noveboracense* isolates, with an average of 3 and 8 mm, respectively. However, no significant difference was observed between the mean degradation halo of *C. chrysophilum* and *C. fioriniae* (Fig. 6e,f).

### Fungicide sensitivity

**Figure 7:**
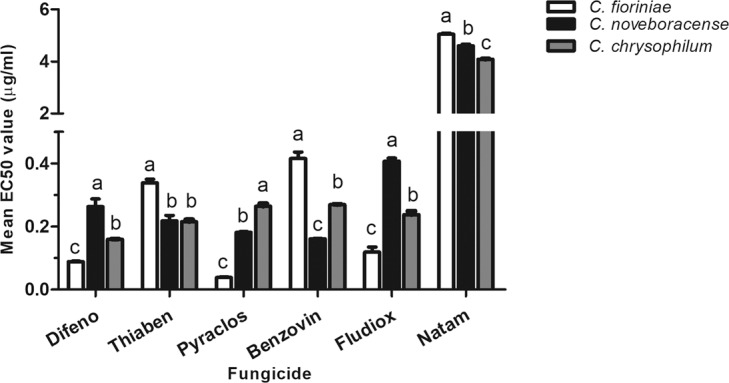
Mean EC_50_ values (µg/ml) of *Colletotrichum fioriniae* (isolates ACFK165, ACFK10, ACFK11, ACFK12, ACFK14, ACFK145, ACFK15, ACFK16, ACFK25, ACFK29, ACFK299, ACFK4, ACFK5, ACFK8, ACFK9, ACFKCM-9, ACFK205, ACFK3 and ACFK6); *Colletotrichum chrysophilum* (isolates AFK17, AFK28, AFK154, AFK18, AFK22, AFK26, AFK30 and AFK31) and *Colletotrichum noveboracense* (isolates AFK220, AFK289, AFK408, AFK423, AFK65 and AFKH109) for fungicides difenoconazole (Difeno), thiabendazole (Tiaben), pyraclostrobin (Pyraclos), benzovindiflupyr (Benzovin), fludioxonil (Fludiox) and Natamycin (Natam). *Colletotrichum* species followed by the same letter were not significantly different based on Bonferroni Comparison Posttest (*p* ≤ 0.05). Error bars represent standard deviation.

Isolates belonging to the three *Colletotrichum* species showed significantly lower sensitivity to natamycin (mean EC_50_ values ranged from 4 to 5 µg/ml) compared to the other fungicides (mean EC_50_ values less than 0.5 µg/ml) (Fig. 7). Relative to *C. noveboracense* and *C.* *chrysophilum*, *C. fioriniae* isolates exhibited greater sensitivity to difenoconazole (EC_50_ value of 0.09 µg/ml), pyraclostrobin (EC_50_ value of 0.04 µg/ml) and fludioxonil (EC_50_ value of 0.12 µg/ml), but had less sensitivity to thiabendazole and benzovindiflupyr with EC_50_ values of 0.4 and 0.3 µg/ml, respectively. With respect to the relative fungicide sensitivity of individual species within the *CGSC*, we found that *C. noveboracense* isolates had significantly higher EC_50_ values in response to the fungicide difenoconazole and fludioxonil compared to *C. chrysophilum*. While all members of the *CGSC* responded similarly to thiabendazole (mean EC_50_ of 0.2 µg/ml), *C. chrysophilum* isolates were significantly less sensitive to pyraclostrobin and benzovindiflupyr (mean EC_50_ values of 0.26 µg/ml) when compared to *C. noveboracense* with mean EC_50_ value of 0.17 µg/ml (Fig. 7).

### Pathogenicity

**Figure 8:**
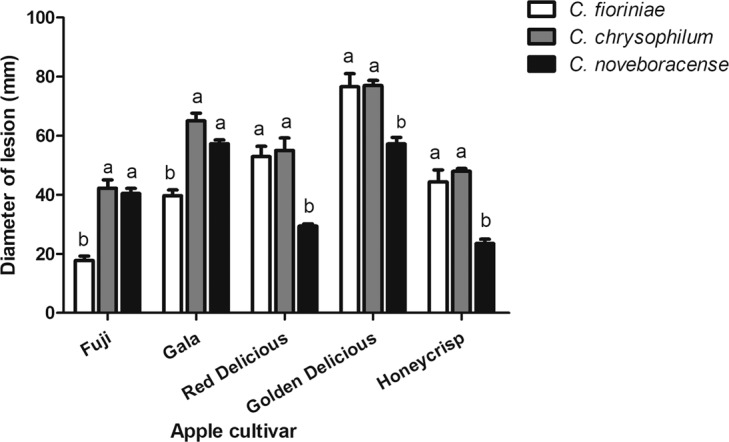
Mean lesion diameter (mm) formed on apple fruit of cultivars ‘Fuji’, ‘Gala’, ‘Red Delicious’, ‘Golden Delicious’ and ‘Honeycrisp’ by *Colletotrichum* species 15 days after inoculation. *Colletotrichum fioriniae* was represented with isolates ACFK145, ACFK15, ACFK16, ACFK205, ACFK29 and ACFK25; *Colletotrichum noveboracense* was represented with isolates AFK220, AFK289, AFK408, AFK423, AFKH109 and AFK65; and *Colletotrichum chrysophilum* was represented with isolates AFK17, AFK28, AFK154, AFK18, AFK31 and AFK22. *Colletotrichum* species followed by the same letter were not significantly different based on Bonferroni Comparison Posttest (*p* ≤ 0.05). Error bars represent standard deviation.

All isolates caused the typical symptoms of bitter rot as light to dark brown and sunken circular lesions on apple fruit of cultivar ‘Honeycrisp’. To meet the requirements of Koch’s postulates, *Colletotrichum* isolates were recovered from inoculated apple fruit and re-identified. Symptoms did not develop on the apple fruit inoculated with agar plugs. In the comprehensive pathogenicity test of selected *Colletotrichum* isolates of each species, the average diameter of lesions varied between the two species complexes and among the species within the *CGSC* complex (Fig. 8). The average diameter of lesions caused by *C. fioriniae* isolates on apple fruit of ‘Fuji’ and ‘Gala’ was significantly smaller, 17.7 and 39.6 mm, respectively, compared to that of produced by *C. chrysophilum* and *C. noveboracense* (Fig. 8). However, in ‘Red Delicious’, ‘Golden Delicious’ and ‘Honeycrisp’, the average lesion diameter caused by *C. fioriniae* isolates were the same as that produced by *C. chrysophilum* isolates (53.2, 76.5 and 44.4 mm) but significantly larger than the lesions developed by *C. noveboracense* (29, 57, 23.5 mm) (Fig. 8).

## Discussion

Effective control of plant diseases caused by *Colletotrichum* species and determination of host specificity and virulence factors are reliant on precise identification and accurate taxonomical delimitation of species boundaries. The assorting of *Colletotrichum* isolates recovered from apple fruit in this study to *CASC* and *C*GSC using a multiplex-PCR^49^, confirmed the reliability and affordability of this method to differentiate between these two species complexes. The *ITS* gene tree placed all the isolates in the *CASC* and *CGSC* with strong support, aligning with previous studies confirming the utility of *ITS* sequencing for classifying *Colletotrichum* isolates at the species complex level^49,52^. In addition, the placement of our isolates in this study into the *CASC* and *CGSC* supports previous evidence that species from these two species complexes are predominantly involved in causing apple bitter rot^16,17,29,43^. Although the application of *ITS* rDNA sequences to identify *Colletotrichum* species was used in studies in the 1990s^70–72^, ITS data are insufficient for identifying species in the *CGSC*^39,40,73^. The multi-locus analyses provided strong resolution and placed the *Colletotrichum* isolates causing bitter rot of apple in New York orchards in *C. fioriniae* clade from *CASC* and *C. chrysophilum* clade from *CGSC*. It also contributed to the identification of a new species in this study, *C. noveboracense*, causing apple bitter rot in New York and Pennsylvania.

In our study, *C. fioriniae* was the dominant species causing bitter rot on apple which is consistent with previous work in Kentucky where *C. fioriniae* was also the most abundant species^17^. *C. fioriniae* causes bitter rot on apple in the US, Korea and Croatia^17,43,74,75^. Seventy percent of isolates recovered from apple orchards in Arkansas, North Carolina and Virginia were identified as *C. acutatum*^76^, the taxon assigned to all *Colletotrichum* strains with acute conidia which later were assigned to *CASC* of over a dozen species^42^.

*C. fructicola* was reported as a causal agent of bitter rot of apple in the USA, Brazil, Korea and Uruguay^24,43^. However, in our work, *C. fructicola* was recovered only from symptomatic apple fruit received from Virginia, not from apple orchards in New York and Pennsylvania. *C. fructicola* was reported to represent the most biological and geographical diversity in the *CGSC*^3^. Its host range and distribution were reported from coffee berries in Thailand, peach in USA, avocado in Australia and apple in USA, Brazil and Korea, to name a few examples of the geographic and host diversity from which this species has been isolated^3^.

First, consisting of two strongly supported monophyletic subclades, *C. ignotum* was described as an endophyte of *Genipa americana, Tetragastris panamensis* and *Theobroma cacao*^52^. This species was later synonymized with *C. fructicola*^3^, with the ex-type of *C. ignotum* and *C. fructicola* nested within the same subclade. Later, it was determined that the second subclade within *C. fructicola* represented an independent evolutionary lineage and was described as *C. chrysophilum*^51^. We detected *C. chrysophilum* for the first time as pathogen on apple in New York and Pennsylvania. It ranks as the second most common species identified in apple orchards in New York, after *C. fioriniae*. In classification of *Colletotrichum* isolates causing anthracnose of peach, using *CAL, GAPDH* and *TUB2*, isolate RR12-3 clustered with *C. fructicola* reference strain ICMP 18645 with bootstrap value 94^49^. By adding five more partial gene sequences, RR12-3 was re-identified as *C. chrysophilum* in our phylogenetic analyses. In addition, we re-identified isolate GA253 as *C. chrysophilum*, that was previously identified as *C. nupharicola*^69^. These findings expand the known host range and geographic distribution of *C. chrysophilum*, which has been identified on cacao and genipa (*Genipa americana*; Panama^52^), fern (*Terpsichore taxifolia*; Puerto Rico^53^), avocado (Israel^69^), peach (South Carolina^49^) and banana (Brazil^51^).

*Colletotrichum noveboracense* was identified as a new species in *Colletotrichum* genus causing apple bitter rot disease in New York and Pennsylvania. The nine *C. noveboracense* isolates from New York and Pennsylvania, as well as a single endophytic isolate from *Juglans nigra* in Oklahoma, formed a distinct clade with high support. In our initial phylogenetic analyses using Bayesian inference, the isolates later attributed to *C. noveboracense* formed a distinct clade in a three-gene multi-locus analysis (*ITS*, *TUB2*, *ApMat*) with full support (BI PP 1.0). Additionally, in our initial Bayesian analysis of seven loci (*ACT, TUB2, CAL, GAPDH, GS, ITS* and *ApMat*) and other different combinations of loci, *C. noveboracense* was sister to *C. nupharicola* (PP = 0.95). *C. nupharicola* is easily distinguished within the *CGSC* in terms of morphology. This host-specific species has very slow growth on PDA and both the length and width of the conidia are much greater than other species in *CGSC*^53,77^. The morphological differences between *C. noveboracense* and *C. nupharicola* prompted us to expand the analysis to include a much larger dataset, include an additional locus (*APN2*) known to provide better resolution in *CGSC*^53^, and evaluate the new clade under GCPSR criteria^78^. This led us to identify these isolates as a strongly supported clade, distinct from other taxa in *CGSC*.

Fungi have developed a plethora of adaptive mechanisms, including extracellular enzyme secretion^79^. In our study, using the skimmed milk agar plates to detect proteolytic activity, it was impossible to observe visible halos of degradation for the assessed isolates. Several possibilities might contribute to the lack of visualization of proteolytic activity in *Colletotrichum* species. First, the ability and the level of protease gene expression in fungi could differ based on the nutrient source used in agar medium. *Aspergillus* isolates showed ability to produce proteases in agar medium supplemented with gelatin and casein as two different sources of protein^80^. Second, the difference in range of pH in culture medium also affects the proteolytic activities^80^. Finally, although the degradation halo indicating the protease activity in *C. fructicola* isolates was detected easily on skimmed milk agar plate^55^, sometimes the detection of the degradation zones is not possible unless a developing agent like bromocresol green dye is used^81^. The three *Colletotrichum* species in our study showed different level of amylolytic, cellulolytic and lipolytic activities. Prior to the present work, only a few studies investigated the enzyme activity of *Colletotrichum* isolates. *C. fructicola* isolates causing bitter rot and leaf spot on apple in Brazil were compared for their ability to produce amylolytic, pectolytic, lipolytic and proteolytic activity, and showed higher amylolytic and pectolytic activity compared to the isolates causing leaf spot, while they were the same in lipolytic and proteolytic activity^55^. Our results show species variation in enzymatic activity, which might be related to variable ability of different *Colletotrichum* species to effectively penetrate and spread in host plant tissues and the higher level of virulence^55,82^. This hypothesis must be further evaluated by investigating the contribution of these enzymes in pathogenicity.

To control bitter rot disease, applications of different fungicides are recommended. We observed statistically different fungicide sensitivity between and within the complexes in our study, which is supported by the previous studies on apple where the *CASC* was more tolerant to thiophanate-methyl, myclobutanil, trifloxystrobin, captan and demethylation inhibitor (DMI) fungicides in comparison to the *CGSC*^17,83–85^. In addition, within the *CASC*, isolates from apple orchards in Brazil showed different levels of sensitivity (25–83%) to mancozeb, thiophanate-methyl and azoxystrobin^62^. All the fungicides in our study showed high mycelial growth inhibition against all the species. Several studies support our findings. Benzovindiflupyr was highly active against mycelial growth of *C. gloeosporioides, C. acutatum, C. cereale* and *C. orbiculare* with EC_50_ values lower than 0.1 μg/ml^86^. Similar efficiency of this fungicide was seen in germination of conidia and germ tube growth of isolates with EC_50_ values 0.1 and 1 μg/ml^86^. Few isolates belonging to *C. fioriniae, C. fructicola* and *C. siamense* were sensitive to fludioxonil, and benzovindiflupyr with EC_50_ values < 0.1 μg/ml and <0.1 to 0.33 μg/ml, respectively^87^. In our study, *in vitro* toxicity of natamycin with an EC_50_ of 5 μg/ml against *Colletotrichum* species was significantly lower than that of the other fungicides with EC_50_ values ranging from 0.04 to 0.4 μg/ml. This is consistent with the previous work in which the toxicity of natamycin against mycelial growth of *C. acutatum* ranged from 0.5 to 1.9 μg/ml in EC_50_ values and was considerably lower compared to fludioxonil, azoxystrobin and cyprodinil^85^. Our data show strong *in vitro* activity of fungicides used in this study and likely would provide effective control of bitter rot in orchards or storages. Although the susceptibility profiles of *Colletotrichum* species against fungicides in apple orchards across the United States are limited, the efficacy of benzovindiflupyr and pyraclostrobin against bitter rot and GLS was reported in two recent trials in North Carolina^88,89^. Future studies should continue to validate the effectiveness of these and other fungicides against apple bitter rot.

In conclusion, three *Colletotrichum* species, *C. fioriniae*, *C. chrysophilum* and a novel species *C. noveboracense*, were identified as the causal agent of apple bitter rot in New York. Also, our study for the first time describes *C. chrysophilum* as the causal agents of bitter rot on apple in Virginia and Pennsylvania and *C. noveboracense* in Pennsylvania. We determined that the three species varied in pathogenicity, enzyme activity and fungicide sensitivity, which are important characteristics for bitter rot management. Our results highlight the significance of accurate identification of *Colletotrichum* species causing bitter rot in apple production regions in order to manage this economically important disease and secure the profitability of apple industry.

## Methods

### Sample collection and fungal isolation

In 2017 and 2018, apple fruit with typical symptoms of bitter rot disease were collected from a variety of apple cultivars in commercial and private apple orchards in the Hudson Valley area, New York (Table 2). Around 400 *Colletotrichum* isolates were obtained from apple fruit disinfected with 5% bleach for 2 min and rinsed with sterile distilled water. After removing the peel around the lesion, three small pieces of fruit pulp cut from the margin of each lesion were placed onto potato dextrose agar (PDA, Difco Laboratories, Detroit, MI, US). Plates were stored at 25 °C in the dark and colonies were purified by hyphal tip method.

**Table 2:** GenBank accession numbers, host and location of *Colletotrichum* strains collected in this study or received from other states to be included in phylogenetic analyses.

Species	Culture/Strain	Host	County/State	GenBank accession number							
				*ACT*	*APN2*	*ApMat*	*CAL*	*GAPDH*	*GS*	*ITS*	*TUB2*
*C. chrysophilum*	AFK154	Apple/Idared	Preston/VA	MN622832	MN653153	MN622868	MN622850	MN689181	MN622841	MN625449	—
	AFK17	Apple/Honeycrisp	Dutchess/NY	MN622831	—	MN622877	MN622859	MN632506	MN622840	MN625458	MN622860
	AFK18	Apple/Honeycrisp	Orange/NY	MN622833	MN653158	MN622876	MN622858	MN632507	MN622842	MN625457	MN622867
	AFK22	Apple/Honeycrisp	Orange/NY	MN622834	MN653157	MN622875	MN622857	MN632505	MN622843	MN625456	MN622866
	AFK26	Apple/Honeycrisp	Columbia/NY	—	MN653156	MN622874	MN622856	MN632508	MN622849	MN625455	—
	AFK28	Apple/Honeycrisp	Orange/NY	MN622835	MN653155	MN622873	MN622855	MN632504	MN622844	MN625454	MN622865
	AFK30	Apple/Honeycrisp	Ulster/NY	MN622836	MN689179	MN622872	MN622854	MN653160	MN622845	MN625453	MN622864
	AFK31	Apple/Honeycrisp	Orange/NY	MN622837	MN653154	MN622871	MN622853	MN653161	MN622846	MN625452	MN622863
	PMAREC-1a	Apple/Idared	Frederick/VA	MN741045	MN790764	MN741076	MN741055	MN741086	MN812242	MN715323	MN741063
	RR12-3^a^	Peach/Un^b^	Saluda/SC	MN741054	MN790772	MN741073	KJ769239	MN741088	MN741102	MN715328	MN741066
	PMCMS-6760	Apple/Un^b^	Lehigh/PA	MN741048	MN790766	MN741079	MN741058	MN741090	MN741104	MN715326	MN741068
	PMKnsl-1	Apple/Un^b^	Adams/PA	Mn741051	MN790769	MN741082	MN741061	MN7741093	MN7411007	MN715327	MN741071
	PMLynd-9a	Apple/Autumn Crisp	Lehigh/PA	MN741052	MN790770	MN741077	MN741062	MN741094	MN741108	MN715332	MN741072
*C. fructicola*	AFK156	Apple/Royal Gala	Carroll/VA	MN622838	MN653159	MN622869	MN622852	MN741095	MN622847	MN625450	MN622862
	RR12-1^a^	Peach	Saluda/SC	MN741053	MN790771	MN741074	KJ769238	—	MN741101	MN715329	MN741065
	PMCrwn-1	Apple/Un^b^	Albemarle/VA	MN741049	MN790767	MN741080	MN741059	MN741091	MN741105	MN715330	MN741069
*C. noveboracense*	AFK220	Apple/McIntosh	Ulster/NY	MN622839	MN653152	MN622870	MN622851	MN689180	MN622848	MN625451	MN622861
	AFK65	Apple/Empire	Columbia/NY	MN701178	MN701188	MN701183	MN701190	MN812243	MN741096	MN708216	MN701196
	AFKH109	Apple/Idared	Columbia/NY	MN640565	MN910262	MN640564	MN640566	MN640567	MN640568	MN646685	MN640569
	AFK289	Apple/McIntosh	Ulster/NY	MN701179	MN701189	MN701182	MN701193	MN741083	MN741097	MN708217	—
	PMBrms-1	Apple/Un^b^	Adams/PA	MN741046	MN790765	MN741075	MN741056	MN741087	MN741100	MN715324	MN741064
	PMCMS-6751	Apple/Un^b^	Lehigh/PA	MN741047	MN790773	MN741078	MN741057	MN741089	MN741103	MN715325	MN741067
	AFK408	Apple/Empire	Ulster/NY	MN701180	MN701187	MN701185	MN701192	MN741084	MN741098	MN708218	MN701195
	AFK423	Apple/Empire	Ulster/NY	MN701181	MN701186	MN701184	MN701191	MN741085	MN741099	MN708219	MN701194
	Coll940	*Juglans nigra*	Cherokee/OK	—	JX145267	JX145325	—	—	—	JX145165	JX145217
	PMEssl-10a	Apple/Un^b^	Lycoming/PA	MN741050	MN790768	MN741081	MN741060	MN741092	MN741106	MN715331	MN741070
*C. fioriniae*	ACFK3	Apple/Empire	Dutchess/NY	—	—	—	—	MN689219	—	MN684827	MN689182
	ACFK4	Apple/Empire	Columbia/NY	—	—	—	—	MN689220	—	MN684828	MN689183
	ACFK5	Apple/Empire	Dutchess/NY	—	—	—	—	MN689221	—	MN684829	MN689184
	ACFK6	Apple/Empire	Orange/NY	—	—	—	—	MN689222	—	MN684830	MN689185
	ACFK8	Apple/Empire	Albany/NY	—	—	—	—	MN689223	—	MN684831	MN689186
	ACFK9	Apple/Empire	Dutchess/NY	—	—	—	—	MN689224	—	MN684832	MN689187
	ACFK10	Apple/Empire	Dutchess/NY	—	—	—	—	MN689225	—	MN684833	MN689188
	ACFK11	Apple/Honeycrisp	Orange/NY	—	—	—	—	MN689226	—	MN684834	MN689189
	ACFK12	Apple/Snap Dragon	Ulster/NY	—	—	—	—	MN689227	—	MN684835	MN689190
	ACFK14	Apple/Honeycrisp	Orange/NY	—	—	—	—	MN689228	—	MN684836	MN689191
	ACFK15	Apple/Honeycrisp	Orange/NY	—	—	—	—	MN689229	—	MN684837	MN689192
	ACFK16	Apple/Empire	Ulster/NY	—	—	—	—	MN689230	—	MN684838	MN689193
	ACFK25	Apple/Honeycrisp	Orange/NY	—	—	—	—	MN689231	—	MN684839	MN689194
	ACFK29	Apple/Honeycrisp	Orange/NY	—	—	—	—	MN689232	—	MN684840	MN689195
	ACFK145	Apple/Empire	Ulster/NY	—	—	—	—	MN689233	—	MN684841	MN689196
	ACFK165	Apple/Gala	Wilkes/NC	—	—	—	—	MN689234	—	MN684842	MN689197
	ACFK205	Apple/Fuji	Suffolk/NY	—	—	—	—	MN689235	—	MN684843	MN689198
	ACFK299	Apple/Nova Easygro	Greene/NY	—	—	—	—	MN689236	—	MN684844	MN689199
	ACFK CM9	Apple/Cider M9	Ulster/NY	—	—	—	—	MN689237	—	MN684845	MN689200

^a^Strains recovered from peach fruit^42^, received from School of Agricultural, Forest and Environmental Sciences, Clemson University

that were sequenced and included in our phylogeny analysis.

^b^Un - cultivar unknown.

### Selection of isolates for molecular analysis

Besides sample collection from New York, we also received bitter-rot infected apple fruit from commercial orchards in Pickerel and Cana, Virginia and Thurmond, North Carolina in 2017 (provided by Virginia Tech Research Station, Winchester, VA) and isolates from Pennsylvania State University's Fruit Research and Extension Center in Biglerville, PA, for identification and comparison. Moreover, two isolates (Cg)RR12-1 and (Cg)RR12-3 identified as *C. fructicola* recovered from peach fruit^49^ were received from School of Agricultural, Forest and Environmental Sciences, Clemson University, SC, for re-identification and comparison. All isolates collected in this study were placed into two morphological typesbased on growth rate, colony texture and color, sporulation and conidial shape on PDA. In total, 44 isolates (31 from New York and 13 from other states) from the two morphologically distinct typeswere selected based on geographical distribution and apple cultivar for identification to the species complex using *ITS* sequencing and multiplex PCR assay, and subsequently to the species level using multi-locus phylogenetic analyses (Table 2). Isolates collected from New York were used for enzyme activity assay, fungicide sensitivity and pathogenicity test.

### Multiplex PCR assay

DNA from mycelia of 7-day-old *Colletotrichum* cultures was extracted using the DNeasy Plant Mini Kit (Qiagen Inc., Valencia, CA, USA) according to the manufacturer’s instructions. A multiplex PCR assay was performed to differentiate isolates of the *CGSC* and *CASC* by partial amplification of the *GAPDH* and *CAL* genes using primer pairs GDF1/C-GAPDH-R, CALF1/Cg-R, and CALF1/Ca-R1^49^. PCR amplifications were carried out in 25 μL volumes containing 10X PCR buffer (includes 20 mM MgCl_2_) (Dream Taq, Thermo Fisher Scientific, Waltham, MA, US), 200 ng of gDNA, 2 mM dNTP, 1 u/μL of Taq DNA polymerase (Dream Taq, Thermo Fisher Scientific, Waltham, MA, US) and 10 μM of each primer using Applied Biosystems 2720 Thermo Cycler (Thermo Fisher Scientific, Waltham, MA, US). Cycling conditions were as follows: initial denaturation of 4 min at 94 °C, followed by 35 cycles of denaturation at 94 °C for 40 s, annealing at 56 °C for 40 s and extension at 72 °C for 1 min, with a final extension at 72 °C for 5 min. PCR products were visualized in 1% (w/v) agarose gels in 1xTAE buffer electrophoresed at 94.1 V for 45 min.

### DNA extraction, PCR amplification and sequencing

DNA was extracted from mycelia of 7-day-old cultures of 44 isolates using the DNeasy Plant Mini Kit (Qiagen Inc, Valencia, CA, US). The partial nucleotide sequences were amplified from eight loci (*ITS, CAL, TUB2, GAPDH, GS, ACT, ApMat* and *APN2)*, and from three loci (*ITS*, *TUB2* and *GAPDH*) for isolates belonging to the *CGSC* and *CASC*, respectively (primer pairs described in Table 3). PCR reactions were performed in 30-μL volumes, including 200 ng of genomic DNA, 10X Dream Taq Green PCR buffer (includes 20 mM MgCl2) (Dream Taq, ﻿Thermo Fisher Scientific, Waltham, MA, US), 2 mM dNTP, 1 u/μL Taq DNA polymerase (Dream Taq, ﻿Thermo Fisher Scientific, Waltham, MA, US) and 10 μM of each primer. Cycling conditions were as follows^3,52^: initial denaturation of 4 min at 95 °C, followed by 35 cycles of denaturation at 95 °C for 30 s, 30 s annealing at 52 (*ITS)*, 59 (*CAL* and *GS)*, 55 (*TUB2)*, 60 (*GAPDH)*, 58 (*ACT* and *ApMat*) and 56 °C (*APN2)*, extension of 45 s at 72 °C, final extension at 72 °C for 10 min. PCR products were examined in 1% (w/v) agarose gels in 1x TAE buffer electrophoresed at 94.1 V for 45 min. PCR product purification and Sanger sequencing were performed by Eurofins Genomics, Louisville, KY, USA.

**Table 3:** List of primers used in this study, sequences and sources.

Product Name	Gene	Primer	Direction	Sequence (5′-3′)
Calmodulin	*CAL*	CL1C	Forward	GAATTCAAGGAGGCCTTCTC^3^
		CL2C	Reverse	CTTCTGCATCATGAGGTGGAC^3^
Glutamine Synthetase	*GS*	GSF	Forward	ATGGCCGAGTACATCTGG^115^
		GSR	Reverse	GAACCGTCGAAGTTCCAC^115^
Glyceraldehyde-3-phosphate dehydrogenase	*GAPDH*	GDF-F	Forward	GCCGTCAACGACCCCTTCATTGA^116^
		GDF-R	Reverse	GGGTGGAGTCGTACTTGAGCATGT^116^
Internal transcribed Spacer	*ITS*	ITS1-F	Forward	CTTGGTCATTTAGAGGAAGTAA^117^
		ITS4	Reverse	TCCTCCGCTTATTGATATGC^118^
β-tubulin 2	*TUB2*	T1	Forward	AACATGCGTGAGATTGTAAGT^119^
		T2	Reverse	TAGTGACCCTTGGCCCAGTTG^119^
Actin	*ACT*	Act512F	Forward	ATGTGCAAGGCCGGTTTCGC^120^
		Act783R	Reverse	TACGAGTCCTTCTGGCCCAT^52^
DNA Lyase	*APN2*	ColDL-F3	Forward	GGGAGAAGCGAACATACCA^52^
		CgDL-R1	Reverse	GCCCGACGAGCAGAGGACGTAGTC^52^
Intergenic spacer and partial mating type (Mat1-2) gene	*ApMat*	CgDL-F6	Forward	AGTGGAGGTGCGGGACGTT^52^
		CgMAT1F2	Reverse	TGATGTATCCCGACTACCG^52^

### Phylogenetic analyses

Consensus sequences were obtained by assembling forward and reverse reads using Geneious Pro v. 11.1.4^90^. In order to confirm the placement of the isolates within species complexes, *ITS* sequences collected from 44 isolates and references from representatives of each of the nine major clades^10^ were used to construct the *ITS* phylogeny. To evaluate the placement of isolates at the species level, the *C. acutatum* phylogeny was constructed using three loci (*ITS, TUB2* and *GAPDH*), whereas the *C. gloeosporioides* phylogeny was constructed using eight loci (*ACT, CAL, GAPDH, GS, ITS, ApMat, APN2* and *TUB2*).

All three phylogenies were constructed using Bayesian inference (BI) and maximum likelihood (ML) approaches. Reference sequences (Supplementary Table S2) were downloaded from GenBank and aligned using MAFFT v7 on-line^91,92^, specifying the G-INS-i iterative refinement strategy. The alignments were trimmed using Gblocks v0.91b^93^ specifying the less stringent criteria. Model selection was conducted using PartitionFinder 2^94^, specifying the Greedy algorithm^95^, “MrBayes” models for BI using PhyML^96^, or “all” models for ML analysis using RAxML^97^ and the AICc metric. Bayesian inference was conducted using MrBayes v3.2.6^98^ implementing the BEAGLE library^99^. For the *ITS* and *C. acutuatum* phylogenies posterior probabilities were estimated using two runs of 2,000,000 generations with 25% burn-in. For the *C. gloeosporioides* phylogeny, 10,000,000 generations were used. ML analysis was conducted using RAxML v8.2.12^97^ specifying 1k bootstrap replications. Clade support was determined by mapping the bootstrap replicates onto the ML best trees using the DendroPy v4.4.0 program SumTrees v4.4.0^100^. BI and ML clade support values were mapped onto the ML best trees using the DendroPy v4.4.0 program SumLabels v2.0.0. Model selection and Bayesian and ML analyses were conducted using the CIPRES Science Gateway^101^. Data handling/formatting was facilitated using AliView v1.26^102^, Mesquite v3.51^103^, SequenceMatrix v1.8^104^ and Geneious v11.1.3 (https://www.geneious.com). Phylogenetic trees were plotted in R v3.4.3^105^ using the Ape package^106^ in the RStudio v1.1.383 environment^107^ and finished using Adobe Illustrator 2020. Sequences generated in this study were deposited in GenBank (Table 2) and the taxonomic novelty in MycoBank. The alignment files and trees were deposited in TreeBase (http://purl.org/phylo/treebase/phylows/study/TB2:S25647).

### Species delimitation

Initial phylogenetic analyses revealed that several *CGSC* isolates collected in this study, in addition to isolate Coll940^53^, clustered together with high support and were distinct from any clade containing the ex-type of any previously described species, suggesting that these isolates may represent a novel lineage. In order to determine whether this new clade formed a distinct phylogenetic lineage, we applied GCPSR^78^. In this approach, a clade is determined to represent an independent evolutionary lineage if the clade satisfies one of two criteria: genealogical concordance or nondiscordance. The genealogical concordance criterion is satisfied if the clade is found well-supported (e.g. both ML and BI analysis ≥70% and ≥0.95, respectively) in most individual gene trees. The nondiscordance criterion is satisfied if the clade is found well-supported in at least one gene tree and members not found strongly supported in contradictory placement (e.g. clustering with the type isolate of another species) in any other individual gene trees.

To apply the GCPSR approach, individual gene trees were constructed for each of the eight genes used in the multi-locus *C. gloeosporioides* phylogeny. Evolutionary model selection and gene tree constructed were as described above, except that 5,000,000 generations were used to infer posterior probabilities for the Bayesian approach. Placement of clade members in each Bayesian and ML tree were evaluated for each individual gene tree.

### Morphological characterization

Colony color, growth rate, conidial shape, length and width of *Colletotrichum* spp. in this study was evaluated by transferring 4-mm diameter plugs from the periphery of 5-day-old cultures, grown at 25 °C in dark, onto PDA and ½ strength PDA. Colony color was described after 7 days of incubation on PDA at 25 °C in dark. Colony growth rate was determined by measuring the colony diameter of each isolate grown on PDA daily over the course of 7 days at 25 °C in dark. To study the morphology of isolates belonging to the novel species, slide culture technique^108^ was used to induce the isolates to produce appressoria. Synthetischer nahrstoffarmer agar (SNA i.e. synthetic nutrient-poor medium)^109^ and oatmeal agar (OMA)^110^ were used to induce sporulation.

Microscopic observations, with 25 measurements per each structure, were viewed with an Olympus BX51 microscope (Olympus Corporation of the Americas, Center Valley, PA, US) using the differential interference contrast (DIC) setting. Statistical analysis was conducted by one-way analyses of variance (ANOVA) using Graph Pad Prism software v5 (San Diego, CA, U.S.A).

### Agar-plate enzyme activity

To perform the qualitative enzyme activity, isolates were grown on PDA at 25 °C for 7 days in the dark. For lipolytic and proteolytic activities, we transferred a mycelial plug from the growing part of each colony onto peptone agar medium (10 g peptone, 5 g NaCl, 0.1 g CaCl_2_ 2H_2_O, 15 g agar, pH 6.0) supplemented with 1% Tween 20^111^ and onto PDA containing 1% soluble skim milk^112^, respectively. After five days of incubation at 25 °C in dark, the size of the clear zone indicating lipolytic and proteolytic activity around each colony was measured in millimeters (mm) using a caliper. For amylolytic activity, isolates were transferred to starch hydrolysis agar medium (pH 7) and kept at 25 °C for 5 days in dark^111^. After flooding with 1 ml of Gram Iodine solution, the clear halo around each colony was measured. Isolates were cultured on PDA supplemented with 0.5% carboxy-methylcellulose (CMC) for 5 days at 25 °C in dark for cellulolytic activity. The plates were treated with 1% Congo red solution and shaken for 15 min. After removing Congo red, cultures were treated with 1 M NaCl and shaken for 15 min. Subsequently, clear zones indicating cellulolytic activity were measured^113^. Three replicates per each isolate was used. Data were analyzed by one-way ANOVA with Graph Pad Prism software v5 (GraphPad Software, San Diego, CA, US).

### Fungicide sensitivity

We evaluated sensitivity of *Colletotrichum* isolates to the technical grade of fungicides pyraclostrobin (Merivon, Pristine, BASF Corporation), difenoconazole (Inspire Super, Syngenta Crop Protection), benzovindiflupyr (Aprovia, Syngenta Crop Protection), thiabendazole (Mertect 340-F, Syngenta Crop Protection), fludioxonil (Scholar, Syngenta Crop Protection) and bio-fungicide natamycin, by using colony growth inhibition assays. We selected these active ingredients as they are registered in the US by the Environmental Protection Agency (EPA) for application in apple orchards or storages. Each isolate was sub-cultured on PDA and grown at 25 °C for 5 days in the dark. Three-mm mycelial disks cut from actively growing parts of each colony were transferred to PDA plates supplemented with pyraclostrobin and thiabendazole at 0, 0.0001, 0.001, 0.01, 0.1, 0.2, 0.5, 1 and 10 μg/ml; difenoconazole at 0, 0.0001, 0.01, 0.02, 0.05, 0.1, 0.2, 0.5, 1 and 10 μg/ml; benzovindiflupyr at 0, 0.0001, 0.001, 0.01, 0.1, 0.2, 0.5, 1, 10 and 20 μg/ml, and fludioxonil at 0, 0.0001, 0.01, 0.03, 0.1, 0.2, 1, 10 and 20 μg/ml. All fungicides were dissolved in acetone. Natamycin was dissolved in methanol and used at 0, 0.2, 1, 2.5, 5, 7.5, 10, 20, 40 μg/ml. Each concentration for each fungicide was replicated five times and the experiment was performed twice. To calculate the EC_50_ values, mean colony diameter and growth rate of each isolate were measured after 5 days incubation at 25 °C in dark^114^. The data were fit to a sigmoidal dose-response curve and EC_50_ values were determined by nonlinear regression using Graph Pad Prism software v5 for Windows OS (GraphPad Software, San Diego, CA, US). Mean EC_50_ of each fungicide for all isolates was compared  using two-way ANOVA with Bonferroni Comparison Posttest using GraphPad Prism v5.

### Pathogenicity assay

Pathogenicity of all isolates was first tested on apple fruit of cultivar ‘Honeycrisp’ to reproduce bitter rot symptoms. Later, six *Colletotrichum* isolates from each species were inoculated on the apple fruit of cultivars ‘Golden Delicious’, ‘Honeycrisp’, ‘Red Delicious’, ‘Fuji’ and ‘Gala’ obtained from a grocery store and washed with detergent and water to ensure that no fungicide residues remain on the surface. Three fruit per each cultivar were disinfected for 2 min in 5% bleach, rinsed twice with sterile distilled water and then wounded with a 3-mm corkborer^43^. Two opposite sides of each fruit were inoculated with 3-mm mycelial plugs of each isolate with aerial mycelia facing the flesh. Control apple fruit received uninoculated agar plugs. Plastic boxes containing inoculated apple fruit placed on moist paper towels were incubated at 25 °C in the dark. Lesion diameter was measured 15 days after inoculation . Data were analyzed by two-way ANOVA with Bonferroni Posttest using Graph Pad Prism software v5 (GraphPad Software, San Diego, CA, US). *P*-values ≤ 0.05 were considered significant. To fulfill Koch’s postulates, strains were re-isolated and morphologically re-identified.

## Supplementary information

Supplementary Information 1.

Supplementary Information 2.

Supplementary Information 3.

Supplementary Information 4.

## Data Availability

Alignments and tree files generated during the current study are available in the TreeBase (Access: http://purl.org/phylo/treebase/phylows/study/TB2:S25647). All sequence data are available in NCBI GenBank following the accession numbers in the manuscript.
