# Author Correction: Identification and characterization of *Colletotrichum* species causing apple bitter rot in New York and description of *C. noveboracense* sp. nov

**DOI:** 10.1038/s41598-020-77484-2

**Published:** 2020-11-24

**Authors:** Fatemeh Khodadadi, Jonathan B. González, Phillip L. Martin, Emily Giroux, Guillaume J. Bilodeau, Kari A. Peter, Vinson P. Doyle, Srđan G. Aćimović

**Affiliations:** 1 Cornell University, Plant Pathology and Plant-Microbe Biology Section, School of Integrative Plant Science, Hudson Valley Research Laboratory, Highland, NY USA; 2 Cornell University, Plant Pathology and Plant-Microbe Biology Section, School of Integrative Plant Science, Ithaca, NY USA; 3 Pennsylvania State University, Department of Plant Pathology and Environmental Microbiology, Fruit Research and Extension Center, Biglerville, PA USA; 4 Pathogen Identification Research Laboratory, Canadian Food Inspection Agency, Ottawa, ON Canada; 5 Louisiana State University AgCenter, Department of Plant Pathology and Crop Physiology, Baton Rouge, LA USA

Correction to: *Scientific Reports* 10.1038/s41598-020-66761-9, published online 06 July 2020

In the original version of this Article, in the Taxonomy section, the typification of the name *Colletotrichum noveboracense* was given as ‘CBS 146410; AFKH109’ rendering the name invalid due to Arts 40.7 and 40.8^1^. The species is validated herein.

*Colletotrichum noveboracense* F. Khodadadi, P.L. Martin, V.P. Doyle, & J.B. Gonzalez & S.G. Aćimović, sp. nov. MB 836581.

For a detailed description see this Article, *Scientific Reports* 10, no. 11043: 7 (2020).

Holotype: The United States of America: New York State, Hudson, from fruit lesion of *Malus domestica* cultivar Idared, July 2017, F. Khodadadi & S.G. Aćimović, BPI 911227.

Ex-holotype culture: CBS 146410; AFKH109.

In Table 2, the GenBank accession numbers for GAPDH and GS of PMKnsl-1 in the manuscript, ‘MN7741093’ and ‘MN7411007’ now read ‘MN741093’ and ‘MN741107’ respectively.

Furthermore, the version of this Article previously published contained lower resolution images for Fig. 2. This has now been corrected in the PDF and HTML versions of the paper.
