# Supplementary figures and images for: Identification and characterization of Colletotrichum species causing apple bitter rot in New York and description of C. noveboracense sp. nov
Source: Sci Rep. 2020 Jul 6;10:11043. doi: 10.1038/s41598-020-66761-9 (PMC7338416; doi:10.1038/s41598-020-66761-9)

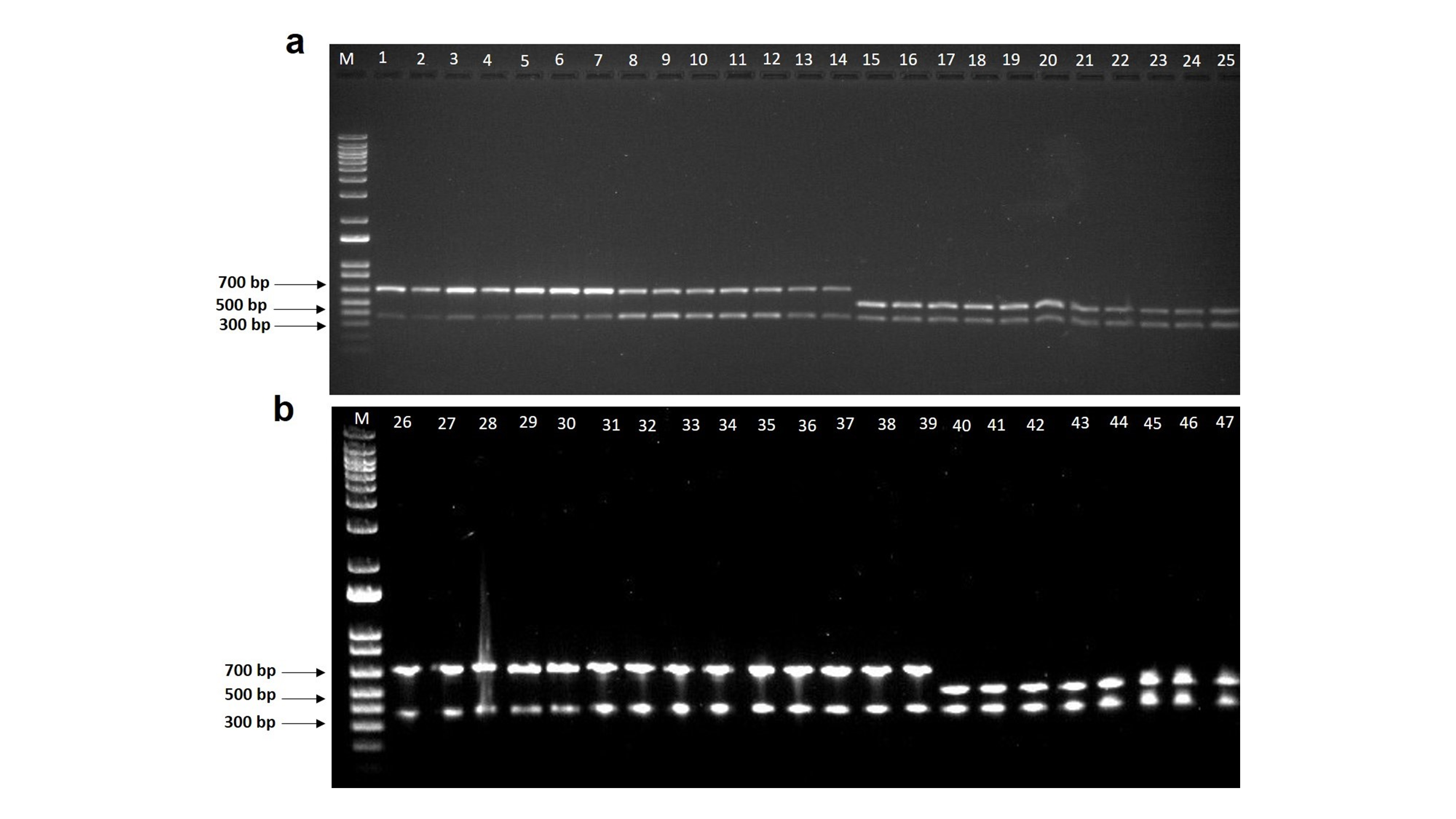

Supplement: Supplementary file 1 — Supplementary Information 1. [file 41598_2020_66761_MOESM1_ESM.tif]

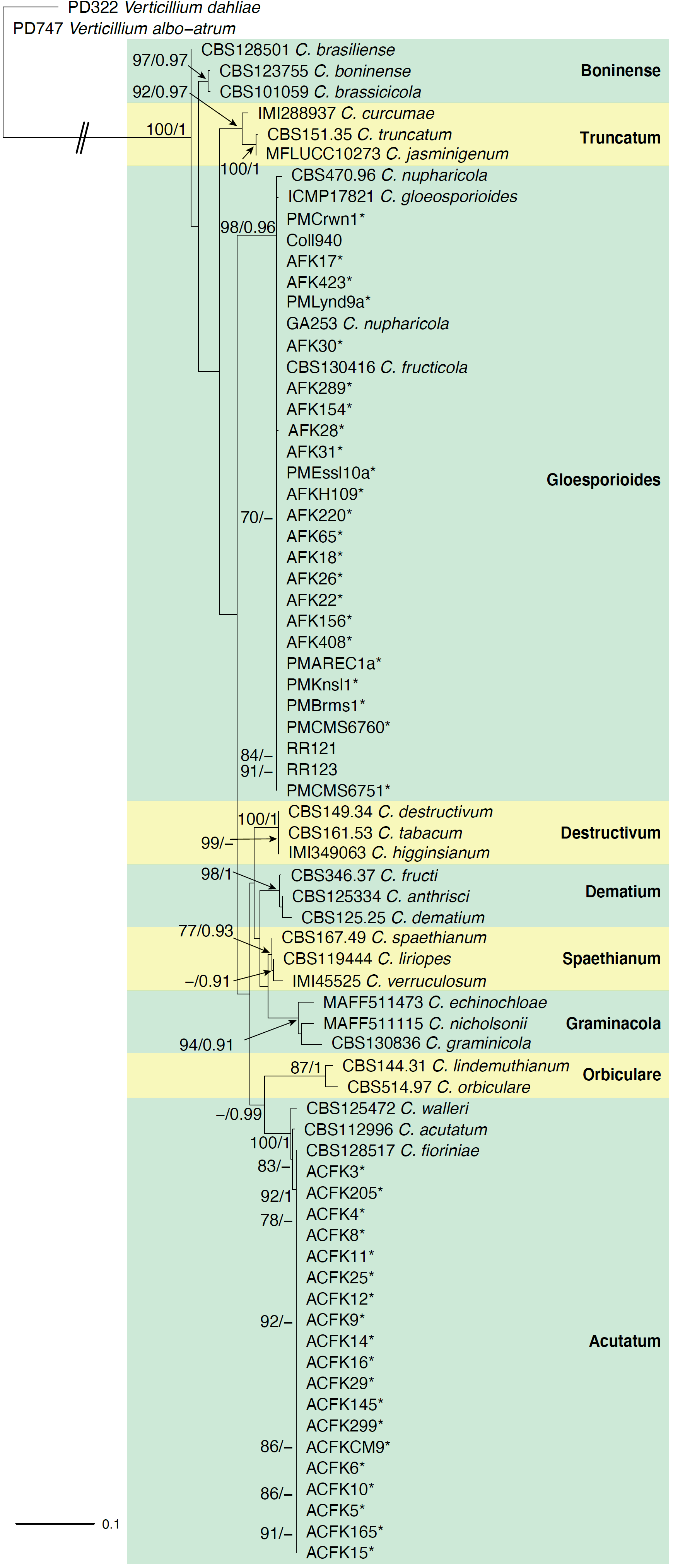

Supplement: Supplementary file 2 — Supplementary Information 2. [file 41598_2020_66761_MOESM2_ESM.tiff]
